# Prognostic value of late gadolinium enhanced MRI in patients underwent coronary artery bypass graft surgery; long term follow up data

**DOI:** 10.1186/1532-429X-17-S1-P109

**Published:** 2015-02-03

**Authors:** Seung-Ah Lee, Yeonyee E Yoon, Jung-Eun Kim, Jin Joo Park, Il-Young Oh, Chang-Hwan Yoon, Jung-Won Suh, Jun Sung Kim, Eun Ju Chun, Sang Il Choi, Young-Suk Cho, Tae-Jin Youn, Cheong Lim, Goo-Young Cho, In-Ho Chae, Kay-Hyun Park, Dong-Ju Choi

**Affiliations:** Cardiology, Cardiovascular Center, Seoul National University Bundang Hospital, Seongnam-si, Korea (the Republic of); Thoracic Surgery, Cardiovascular Center, Seoul National University Bundang Hospital, Seongnam-si, Korea (the Republic of); Radiology, Cardiovascular Center, Seoul National University Bundang Hospital, Seongnam-si, Korea (the Republic of)

## Background

It has not been well evaluated whether late gadolinium enhanced (LGE)-MRI can provide prognostic information in patients who underwent coronary artery bypass graft (CABG) surgery, especially in patients with preserved left ventricular ejection fraction (LVEF). Further, long-term follow-up data after MRI and CABG surgery are not available. The purpose of this study was to evaluate the impact of myocardial viability assessment by LGE-MRI on prognosis in patients who underwent CABG surgery.

## Methods

A total 145 consecutive patients (age 64.1 ± 8.8 years; male 28%) with who underwent cine- and LGE-MRI before CABG surgery were investigated. The presence and transmurality (0, absent; 1, 1-25%; 2, 26-50%; 3, 51-75%; 4, 76-100%) of LGE was visually determined using a standard 17-segment AHA model. LGE score was defined as the sum of each segment scale. The outcome measures were adverse cardiac events (cardiac death, nonfatal myocardial infarction [MI], heart failure [HF] and unstable angina) and cardiovascular events (adverse cardiac events and stroke).

## Results

**Figure 1:**
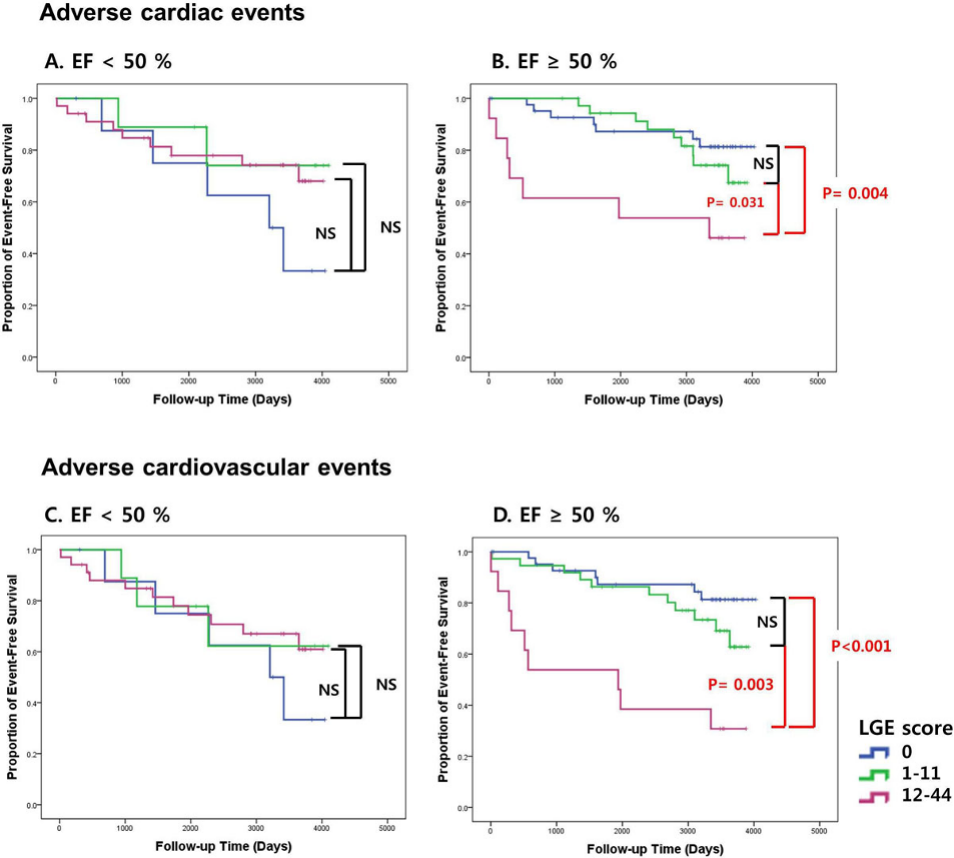
Kaplan-Meier event-free survival curves stratified by LGE score.

LGE was observed in 93 of 145 patients (64.1%). Average number of segments with LGE was 3.3 ± 3.6, and LGE score was 7.8 ± 9.1 in the entire study cohort. During a median follow-up period of 8.7 year (Interquartile range, 7.4 to 9.5 years), 47 patients (32.4%) experienced adverse cardiovascular events (10 cardiovascular deaths, 9 MIs, 9 HFs, 18 unstable anginas and 8 vascular events). The analysis was carried out by strata according to LGE score (LGE score = 0 [n = 52]; 1-11 [n = 46]; 12-44 [n = 47]). For both adverse cardiac and cardiovascular events, high LGE score of 12-44 were significantly associated with worse event-free survival compared to those without LGE and those with LGE score of 1-11, in a subgroup with preserved LVEF ≥ 50%. In contrast, high LGE score was not associated with adverse cardiac and cardiovascular events in a subgroup with decreased LVEF < 50%. (Figure 1).

## Conclusions

Myocardial viability assessment by LGE-MRI has impact on prognosis after CABG surgery in patients with preserved LVEF. This observation may extend the indication of viability assessment to those without severe LV dysfunction before CABG surgery.

## Funding

N/A.

